# Lithium-Induced Nephropathy in Bipolar Disorder: A Clinical Concern

**DOI:** 10.1192/j.eurpsy.2026.10421

**Published:** 2026-07-31

**Authors:** J. Marta, M. Magalhães, S. Mendes, M. A. Aleixo

**Affiliations:** Setúbal Hospital Center, Setúbal, Portugal

## Abstract

**Introduction:**

Lithium remains a cornerstone in the treatment of Bipolar Disorder (BD), as the first mood stabilizer introduced and still considered a first-line option. Despite its proven efficacy, lithium therapy is associated with a narrow therapeutic window and potential long-term adverse effects, particularly nephrotoxicity.

**Objectives:**

This review aims to synthesize current evidence on lithium-induced nephropathy in BD, identify key risk factors, clinical and histological features, and prognostic considerations.

**Methods:**

A non-systematic literature review was conducted using PubMed with the keywords “lithium”, “nephropathy” and “bipolar disorder”.

**Results:**

The prevalence of renal dysfunction among patients on long-term lithium therapy is approximately 25%, with women under 60 showing the highest risk (57-63%). Between 15-25% of patients may experience a gradual decline in Glomerular Filtration Rate (GFR), potentially leading to end-stage renal disease (ERD) after decades of exposure. Risk factors include treatment duration, cumulative dose, episodes of acute intoxication, advanced age, comorbidities, and concomitant use of antipsychotics or diuretics. The clinical presentation is typically insidious, with polyuria, proteinuria, nephrogenic diabetes insipidus and progressive decline in GFR. Histological findings include microcyst formation in both cortex and medulla, interstitial fibrosis, and focal segmental glomerulosclerosis in severe cases. Pathophysiology involves lithium entry through renal sodium channels, causing tubular injury and impaired water regulation. Stopping lithium may stabilize or slow disease progression, particularly in early stages, but full reversibility is rare. Recovery is more likely when baseline GFR exceeds 30mL/min, with stabilization usually occurring within 12-24 months after discontinuation. No specific treatment exists beyond lithium withdrawal and management of complications, although amiloride may reduce polyuria, without robust evidence for preventing chronic nephropathy. Prognosis is influenced by baseline renal function, proteinuria burden, comorbidities, and histological severity.

**Conclusions:**

Individualized risk stratification and careful dose adjustment are essential, aiming for serum levels at the lower therapeutic range (0.4-0.6mEq/L). Single daily dosing regimens appear to reduce nephrotoxicity when compared with divided doses. Regular monitoring of renal function and lithium serum levels is crucial, and treatment discontinuation should be considered in cases of progressive renal decline. Clinical decision-making must balance psychiatric benefit with nephrotoxic risk, highlighting the importance of early detection, preventive measures, and shared decision-making in long-term management.

**Disclosure of Interest:**

None Declared

